# Using machine learning to predict acute myocardial infarction and ischemic heart disease in primary care cardiovascular patients

**DOI:** 10.1371/journal.pone.0307099

**Published:** 2024-07-18

**Authors:** N. Salet, A. Gökdemir, J. Preijde, C. H. van Heck, F. Eijkenaar

**Affiliations:** 1 Erasmus School of Health Policy & Management, Erasmus University Rotterdam, Rotterdam, The Netherlands; 2 Esculine b.v., Capelle aan den IJssel, South Holland, The Netherlands; 3 DrechtDokters, Hendrik-Ido-Ambacht, South Holland, The Netherlands; HT Ong Heart Clinic, MALAYSIA

## Abstract

**Background:**

Early recognition, which preferably happens in primary care, is the most important tool to combat cardiovascular disease (CVD). This study aims to predict acute myocardial infarction (AMI) and ischemic heart disease (IHD) using Machine Learning (ML) in primary care cardiovascular patients. We compare the ML-models’ performance with that of the common SMART algorithm and discuss clinical implications.

**Methods and results:**

Patient-level medical record data (n = 13,218) collected between 2011–2021 from 90 GP-practices were used to construct two random forest models (one for AMI and one for IHD) as well as a linear model based on the SMART risk prediction algorithm as a suitable comparator. The data contained patient-level predictors, including demographics, procedures, medications, biometrics, and diagnosis. Temporal cross-validation was used to assess performance. Furthermore, predictors that contributed most to the ML-models’ accuracy were identified.

The ML-model predicting AMI had an accuracy of 0.97, a sensitivity of 0.67, a specificity of 1.00 and a precision of 0.99. The AUC was 0.96 and the Brier score was 0.03. The IHD-model had similar performance. In both ML-models anticoagulants/antiplatelet use, systolic blood pressure, mean blood glucose, and eGFR contributed most to model accuracy. For both outcomes, the SMART algorithm was substantially outperformed by ML on all metrics.

**Conclusion:**

Our findings underline the potential of using ML for CVD prediction purposes in primary care, although the interpretation of predictors can be difficult. Clinicians, patients, and researchers might benefit from transitioning to using ML-models in support of individualized predictions by primary care physicians and subsequent (secondary) prevention.

## Introduction

Over the past 20 years, cardiovascular disease (CVD) has been the most common cause of death worldwide [1]. Most of this mortality can be attributed to ischemic heart disease, accounting for around 16% of fatalities. In addition, CVD is also among the most costly diseases worldwide [2, 3]. A recent study using data from England and Wales found that a reduction of 1% in the number of cardiovascular events would lead to an estimated 34 million euros of estimated savings [4]. The impact of CVD can, however, be mitigated by early recognition of at-risk patients and subsequent application of (secondary) preventive measures such as changes in lifestyle or pharmaceutical intervention [5]. Therefore, great effort is being put in CVD prevention strategies [6]. Primary care is an appropriate setting for many of such strategies, especially in the many countries where general practitioners (GPs) function as gatekeepers to secondary care.

The use of prediction models for identification of cardiovascular risk in primary care can play a vital role in CVD prevention and risk management activities. Although the importance of early recognition of cardiovascular risk is evident, accurate risk prediction remains complex. Presently, several CVD risk prediction algorithms are available for GPs. Examples are the Framingham risk score, SCORE, SMART and QRISK algorithms [7]. Although these algorithms are rarely used directly by general practitioners (GPs) themselves, they sometimes are incorporated in intervention guidelines [5]. These algorithms are based on linear or logistic regression models which generally attempt to estimate associations and their statistical significance between patient-level predictors and CVD-related outcomes. These results are then used in guidelines to classify patient risk and distribute patients over cardiovascular risk categories. Recently, artificial intelligence techniques, particularly machine learning (ML), have shown promising results in terms of their ability to predict patient-level risks with high sensitivity, specificity, precision, and accuracy [8–13]. As such, these methods may be more suitable for risk prediction purposes than algorithms based on conventional regression modelling [14, 15]. While conventional regression models have been fundamental in clinical risk prediction, the shift towards machine learning models is justified by their ability to better handle complex, high-dimensional data, automatically model interactions, and provide higher prediction accuracy and adaptability [16]. Nevertheless, direct comparisons of ML with existing CVD risk prediction algorithms on the same data remain rare [14, 15].

A recent systematic literature review on CVD prediction models highlighted several shortcomings of existing research on CVD risk prediction [5]. First, many prediction models focus on the general population and not on distinct subpopulations such as cardiovascular patients in primary care. Second, existing prediction models generally focus on predicting CVD in general, instead of on distinct events or conditions such as acute myocardial infarction (AMI) or ischemic heart disease (IHD). Making such distinctions seems important from a prevention standpoint because risk factors may differ between AMI and IHD, which would justify separate prediction models. Third, models tend to be inadequately reported, insufficiently validated, and lack information on usefulness for individual-level risk prediction in clinical practice. For example, studies often do not report which risk factors contributed most to the prediction performance and therefore lack crucial information on targets for intervention. Finally, studies typically do not present head-to-head comparisons of the performance of different models in specific settings, information that is needed for building better CVD prediction models.

The contribution of our study is threefold. First, we use ML to develop CVD prediction models using data from primary care cardiovascular patient records containing International Classification of Primary Care (ICPC) diagnose codes as well as a large variety of patient-level predictors. Specifically, we built two random forest models: one model for predicting acute myocardial infarction (AMI) and a second model to predict symptomatic ischemic heart disease (IHD) with angina pectoris. We deliberately focused on symptomatic IHD because of the importance of monitoring the degree to which the disease stabilizes, which would also reduce the risk of more severe heart disease. Second, the performance of both ML-models is directly compared to the performance of the Second Manifestations of ARTerial disease (SMART) algorithm in predicting the two outcomes. The SMART algorithm is a risk prediction model that was developed for patients with manifest cardiovascular or vascular disease, aiming to predict major cardiovascular events considering their elevated risk [7, 17]. As such, in the context of (secondary) prevention in this study, it is the most suitable comparator for new methods, such as ML. In essence, the SMART algorithm is a rule-based approach that relies on predefined criteria, while ML models learn patterns directly from data. Finally, we identify the predictors that contribute most to the accuracy of our ML models and reflect on the extent to which these models can aid clinical decision-making and under which conditions.

## Methods

### Data and study population

We used anonymized electronic health record (EHR) data collected between 01-01-2011 and 31-03- 2021 on primary care cardiovascular patients who were enrolled in a cardiovascular risk management (CVRM) program in the Netherlands. The provided dataset was anonymized and kept in an encrypted, access-controlled environment. This study was based on legally obtained, existing and anonymous data. Consent from DrechtDokters and EscuLine was acquired for scientific use of data prior to the conduction of this study. All methods were carried out in full accordance with privacy regulations and guidelines.

Consequently, follow-up stops 31^st^ of March 2021, and the duration of follow-up may vary between patients depending on when they entered or left the program. In general, patients are eligible for enrolment in a CVRM program if they have an elevated risk of CVD. This risk can be determined based on several factors, such as high blood pressure, high cholesterol levels, smoking, diabetes, family history of CVD, obesity, or a previous diagnosis of CVD [18]. In CVRM programs, GPs, practice nurses, physiotherapists, and dietitians work together to offer guidance and support to patients, aiming to prevent further cardiovascular issues. The data were provided by Drechtdokters, a Dutch non-profit organization representing more than 90 GP practices that aim to provide value-driven care to patients in its region. The dataset contains various patient-level variables including demographic characteristics (age, sex), medication use, biometrics (e.g., vital signs, laboratory test results), diagnosis history, and lifestyle-related factors. All included patients were enrolled in a CVRM program and had at least one of the relevant International Classification of Primary Care (ICPC) diagnosis codes that are commonly associated with increased risk of CVD (see S1 Appendix). All disease definitions were based on registered ICPC diagnosis codes. These definitions were subjected to strict validation processes to enhance data reliability.

**Fig 1:**
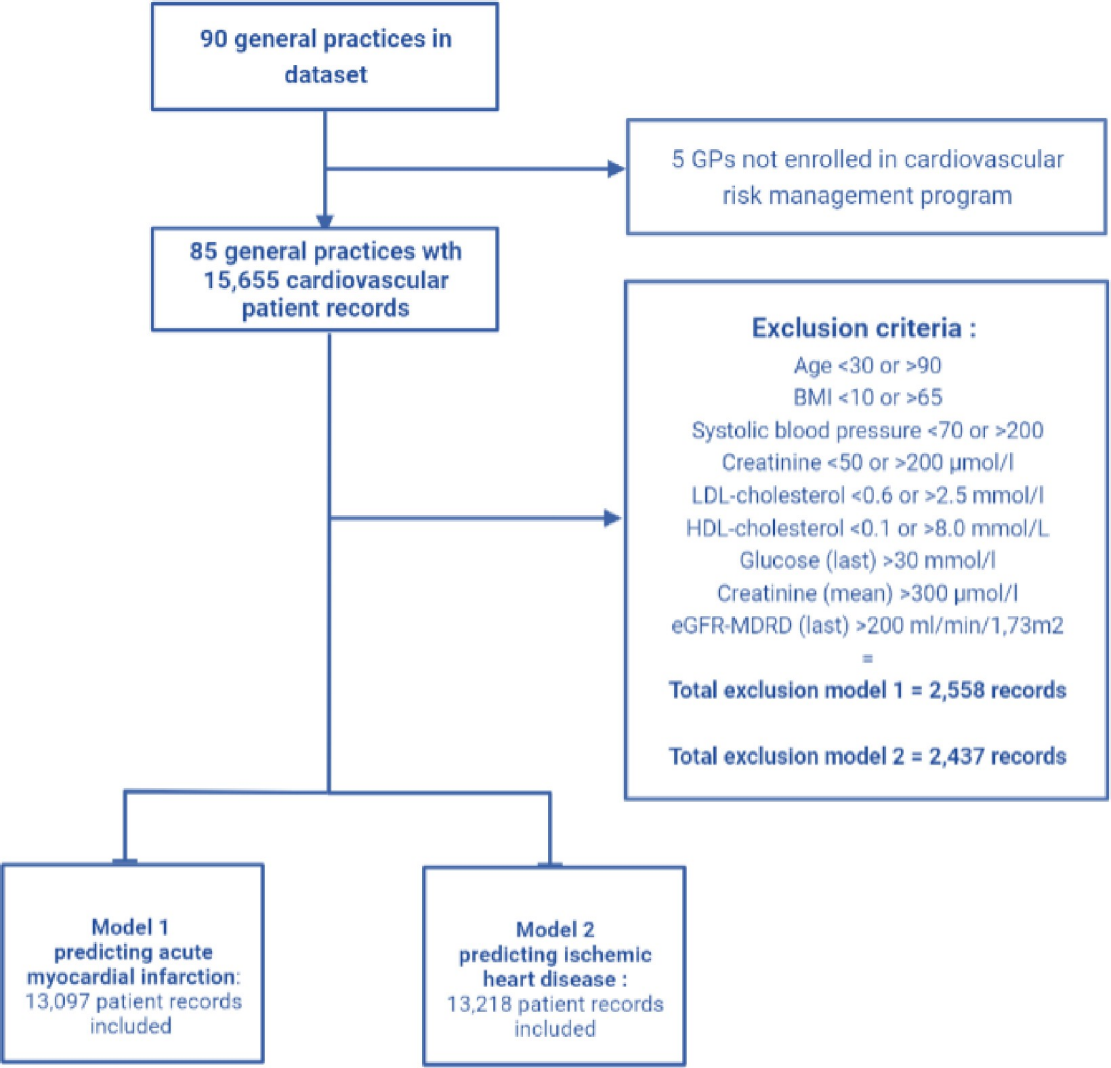
Flowchart of study population, selection procedure and exclusion criteria. The difference in patient records between the two models was caused by differences in the age at which patients were diagnosed with cardiovascular disease and were enrolled in a cardiovascular risk program.

Separate datasets were built for each ML model because of differences in the age at which patients were diagnosed with CVD and enrolled in a CVRM program. As a result, there was a small difference in the number of included patient records in the two ML models. In total, after data imputation (see below) and applying exclusion criteria (see Fig 1) 13,097 cardiovascular patient records were included in the model predicting AMI. The second model, predicting IHD with angina pectoris, included 13,218 patient records. The SMART algorithm was also applied to these two patient populations and its performance was directly compared to the performance of the two ML models [19]. Rather than predicting long-term risk of cardiovascular events in CVD patients, we used the SMART algorithm to compare our ML models with in terms of their performance in predicting the two outcomes (i.e., yes/no AMI and yes/no symptomatic IHD with angina). It is important to acknowledge that no prediction models validated specifically for primary care cardiovascular patients exist for this population. In this context, the SMART algorithm is the best available benchmark for making comparisons.

Patients with over 50% missing predictors (either missing completely at random or at random) were excluded (see Fig 1). For the remaining patients, missing values were imputed using the ‘k-Nearest neighbours algorithm’[20]. The k-nearest neighbors method was chosen due to its ability for handling missing values without relying on strong assumptions and ensuring contextually relevant imputations that minimize bias in the dataset. This is a method for estimating plausible values by using the k most similar available data points, in our case five. The algorithm finds the ‘nearest neighbours’ by measuring the Euclidian distance between known values of measurements using the values of similar patients [21]. The resulting imputed value is the mean value weighted by the distance to the five nearest neighbours. By doing so, this technique estimates plausible values for the missing data points. In total, 18.1% of datapoints were imputed for model 1 and 2.

### Predictors

We selected predictors based on literature and data availability. Given their relevance to CVD in general, the same predictors were used for both ML models. First, several non-modifiable factors were included. It is well-established that both higher age and the female sex are associated with increased risk of CVD [5, 22, 23]. Age and sex are therefore widely used in CVD prediction models, including our models (with age defined as the age at the time of the initial CVD diagnosis). In addition, chronic obstructive pulmonary disease (COPD) [24, 25], bronchial asthma [26], and diabetes mellitus (DM) [27] are well-known predictors for increased CVD risk, and were therefore included. Furthermore, we incorporated various pre-existing vascular diseases as independent predictors in our models due to their well-established association with an elevated risk of CVD. These diseases include ischemic heart disease with or without angina (included for the AMI model only), coronary sclerosis, transient ischemic attack (TIA), stroke, intermittent claudication (vascular claudication), aortic aneurysm, as well as prior AMI or IHD [28].

Second, we included several modifiable predictors. Both systolic and diastolic blood pressure can impact the risk of cardiovascular events [29], and elevated HDL- and LDL-cholesterol are associated with increased risk of CVD and were therefore included [30]. Since the severity of DM is modifiable and given that high blood glucose levels are associated with higher risk of CVD, sober venous blood glucose levels were also included [31]. In addition, kidney function (eGFR: estimated glomerular filtration rate) and creatinine values are known independent predictors of CVD, and were therefore added as well [32] Furthermore, lifestyle-related factors including smoking status, body mass index (BMI) and degree of physical activity are known risk factors for CVD and were thus included [33, 34] Also, the use of asthma medications (S2 Appendix Table 1) has been found to be associated with increased risk of CVD while the use of anticoagulants/antiplatelets and antiplatelets (S2 Appendix Table 2) decreases CVD risk. Both factors were therefore included in de models [35].

**Table 1:** Descriptive statistics of study population before data imputation, by diagnosis. * See Appendix 2 for a list of included medications.

	Description	Model 1 ‐ Predicting acute myocardial infarction	Model 2 ‐ Predicting ischemic heart disease
**General practices and patients**	Number of general practices	85	85
	Number of patients	13,097	13,218
	Age in years, mean (SD)	70.6 (11.5)	70.7 (11.1)
	Male sex, n (%)	7,764 (59.3%)	7821 (59.2%)
**Comorbidities**	Ischemic heart disease with angina (%)	9.3	1.3
	Stable angina pectoris (%)	1.7	0.3
	Unstable angina pectoris (%)	1.7	0.3
	Myocardial infarction (%)	1.9	1.6
	Ischemic heart disease (%)	2.6	0.4
	Coronary sclerosis (%)	2.7	2.7
	Prior myocardial infarction (%)	1.4	1.4
	Transient ischemic attack (TIA) (%)	13.4	13.2
	Cerebrovascular accident (CVA) (%)	3.2	3.1
	Intermittent claudication (%)	5.9	5.8
	Aortic aneurysm (%)	3.4	3.4
	Chronic obstructive pulmonary disease (COPD) (%)	8.2	8.1
	Bronchial Asthma (%)	5.2	5.1
	Diabetes mellitus type 2 (%)	4.5	4.5
	Rheumatoid arthritis (RA) (%)	0.6	0.6
Medications*	Use of (anti-inflammatory) asthma medications (%)	24.2	24.2
	Use of anticoagulants/antiplatelets (%)	71.0	61.8
**Degree of physical activity (last)**	0 (inactive) (%)	2.3	2.2
	1–4 (below norm) (%)	23.7	23.9
	5 or higher (conform norm) (%)	73.2	73.0
	Unknown (%)	0.9	1.1
**Smoking status (last)**	Active smoker (%)	19.0	16.4
	Never smoked (%)	33.3	33.2
	Former smoker (%)	41.9	44.9
	Unknown (%)	5.8	5.6
**Degree of physical activity (max)**	0 (inactive)	1.7	1.7
	1–4 (below norm)	22.4	19.0
	5 or higher (conform norm)	74.6	78.4
	Unknown	1.2	1.0
**Smoking status (max)**	Current smoker	14.8	15.5
	Never smoked	34.6	38.6
	Former smoker	46.6	42.2
	Unknown	4.0	3.8
**Biometrics**	Sober venous blood glucose, mean (SD) (last)	5.4 (0.9)	5.4 (0.9)
	Sober venous blood glucose, mean (SD) (mean)	5.3 (0.6)	5.4 (0.6)
	eGFR-CKD-EPI formula, mean (SD) (last)	70.2 (13.5)	69.9 (13.3)
	eGFR-CKD-EPI formula, mean (SD) (mean)	70.7 (11.8)	71.3 (11.8)
	eGFR-MDRD formula, mean (SD) (last)	59.4 (5.6)	59.2 (5.5)
	Creatinine, mean (SD) (last)	87,4 (20.7)	86.9 (20.4)
	Creatinine, mean (SD) (mean)	85.0 (16.3)	85.1 (16.2)
	LDL-cholesterol, mean (SD) (last)	2.8 (0.9)	2.7 (0.9)
	LDL-cholesterol, mean (SD) (mean)	2.7 (0.7)	2.7 (0.7)
	HDL-cholesterol, mean (SD) (last)	1.4 (0.4)	1.4 (0.3)
	HDL-cholesterol, mean (SD) (mean)	1.3 (0.3)	1.3 (0.3)
	BMI, mean (SD) (last)	27.4 (4.1)	27.5 (4.2)
	BMI, mean (SD) (mean)	27.2 (3.6)	27.3 (3.5)
	Diastolic blood pressure, mean (SD) (last)	78.8 (9.0)	78.3 (8.8)
	Diastolic blood pressure, mean (SD) (mean)	77.8 (6.9)	77.4 (6.5)
	Systolic blood pressure, mean (SD) (last)	136.7 (15.2)	136.0 (15.1)
	Systolic blood pressure, mean (SD) (mean)	135.2 (11.5)	134.8 (11.7)

**Table 2:** Cross-validated performance metrics for models predicting AMI (mean of folds).

Performance metric	Machine learning model 1	SMART 1
Accuracy	0.96	0.67
Sensitivity	0.67	0.03
Specificity	1.00	0.80
Precision	1.00	0.03
AUC/c-statistic	0.96	0.42
Brier-score	0.04	0.33

When the data contained multiple measurements for single patients over time (e.g., multiple measurements of blood pressure), these measurements were used to define two variables, both of which were included: ‘last measurement’ (i.e., the most recent measurement) and ‘measurement mean’ (i.e., the mean of all available measurements). Because our ML models already consider potential interactions between predictors, no further analyses to determine interactions were conducted.

The SMART algorithm was then replicated, which means that the following variables have been included: age, sex, years since first cardiovascular event, systolic blood pressure, creatinine, HDL-cholesterol, LDL-cholesterol, smoking status, use of anticoagulants/antiplatelets, DM, and atherosclerotic vascular disease were included. Data on C-reactive protein (CRP) was unfortunately unavailable for most patients, and we therefore used the population mean (= 2.2 mg/L) when applicable, following the same methodology as employed in the SMART algorithm [36].

### Statistical analysis

Our goal was to investigate and illustrate the potential of ML for CVD prediction in primary care in a clear and comprehensible manner. We selected the random forest method because these are relatively computationally efficient compared to more complex models like gradient boosting or neural networks, especially when dealing with larger datasets. Given the scope and resources, we prioritized models that are more straightforward to implement and interpret, ensuring that our findings could be translated into clinical practice more easily. This method has shown promising results in terms of predictive accuracy and limited overfitting, particularly in studies on CVD prediction [14, 15, 37, 38]. It is a ML method that is used for classification or regression problems and is generally found to be suitable for both categorical and continuous outcomes [14, 15, 39]. In essence, the method combines multiple decision trees, which can be interpreted as visual representations of different potential paths leading to a specific objective. The fundamental concept behind it is that variables that depend on each other are divided into subsets, also known as branches, by identifying the best possible split based on predictor values. The random forest algorithm classifies observations by passing them through each decision tree. With this information, the frequencies of outcomes of the model (the predicted class) can be calculated. The predicted class with the highest frequency represents the final category in which an observation is classified. This ‘majority voting’ is expected to result in stronger prediction and aims to optimize classification [40]. The performance of random forest models depends on the number of decision trees, with the optimal number of trees varying depending on the specific problem, dataset, and available computational resources. Increasing the number of trees can reduce variance, making the model less sensitive to changes in the data and improving its generalizability. However, there is a diminishing return on variance reduction beyond a certain number of trees. The number of trees in our models was iteratively set at 500 [20, 21].

We used grid search and random search for hyperparameter optimization, but these techniques ultimately provided no or minimal improvement in model performance compared to the default settings. Therefore, we opted to used the default settings.

To prevent overfitting and mitigate the potential impact of differences in the timing of patient enrolment and exit from the program, we used 5-fold temporal cross-validation. Cross-validation is particularly useful when dealing with time-dependent health data (as is the case for our data) and helps to create accurate assessments of how well a model performs in practical, real-world scenarios. Separately for the AMI and IHD sample, we randomly divided the data into five equal-sized parts or ‘folds’ [41–44]. Four parts were used for training our models and one part for validation (i.e., for generating predictions and calculating performance measures). This process was repeated for each fold, in such a way that each fold functioned as validation set once. In other words, in each of the iterations, each model was estimated four times on four different sets of training data (in total containing 80% of observations but in a different composition, distributed over 4 folds each containing 20% of the data) and tested on a different validation set (the fifth fold, containing the remaining 20% of observations). To evaluate model skill, we used the averages of the performance metrics calculated on the ‘rotating’ validation sets (for each iteration of cross-validation there is a different validation set). Predicted values for the observations in each validation set were generated using the models trained on the training set.

For the calculation of several performance metrics (explained below), we created confusion matrices by comparing predicted values with the actual values of the outcome variables in the validation data. A confusion matrix has four cells: true positives (TP), true negatives (TN), false positives (FP) and false negatives (FN). In this study, the occurrence of AMI or IHD is considered as a positive case. Because fewer patients had either AMI or IHD than not (i.e. negative cases outweigh positive cases), we used stratified sampling based on outcome (i.e., AMI or IHD) to prevent unequal distribution of positive and negative cases between the training and validation data [45].

We evaluated model skill using six performance metrics. First, accuracy is the proportion of correct predictions and calculated as $\frac{\left(\text{TP}+\text{TN}\right)}{\left(\text{TP}+\text{TN}+\text{FP}+\text{FN}\right)}$. Second, sensitivity (i.e., true positive rate) is the percentage of positive cases that were correctly classified as such and was calculated as $\frac{\text{TP}}{\left(\text{TP}+\text{FN}\right)}$. Similarly, specificity (i.e., true negative rate) is the percentage of negative cases that were classified as such and was calculated as $\frac{\text{TN}}{\left(\text{TN}+\text{FP}\right)}$. In the context of this study, false positive predictions may lead to unnecessary referrals to hospitals whereas false negative predictions may lead to underdiagnosis and harmful consequences for patients. Therefore, high sensitivity is preferable over high specificity. Fourth, precision is proportion of true positive predictions (TP) out of all positive predictions was calculated as $\frac{\text{TP}}{\left(\text{TP}+\text{FP}\right)}$. It reflects the degree to which the same results can be expected upon repeated measurement. Considering the anticipated class imbalance in the data, which means that positive and negative cases are not represented equally, relying solely on accuracy, sensitivity, specificity, and precision may lead to distorted insights. For instance, if most of the data (e.g., 90%) belongs to class A, a model could achieve a high accuracy (e.g., 90%) by simply assigning every observation to class A. This would not provide meaningful insights. Additionally, such a model may introduce bias towards the overrepresented class (in our case, the negative cases). Therefore, we also calculated the area under the receiver operating characteristic curve (AUC or c-statistic) as well as the Brier score. The AUC is a measure of discrimination and classifies model performance at various thresholds. It is acquired by plotting the true positive rate (TPR, i.e., sensitivity) against the false positive rate (FPR). AUC values were interpreted as poor (<0.7), fair (0.7–0.8), good (0.8–0.9) or excellent (≥ 0.9) discriminative ability [46]. The Brier score (range 0 = perfect and > = 0.25 = non-informative) indicates how well a model’s predicted probabilities align with the true outcomes [47]. No further data balancing techniques were used because random forest models are inherently robust to class imbalances. They handle possible imbalanced data by assigning higher weights to minority class instances during training, thereby mitigating the impact of class imbalance on model performance.

For each model, we then identified the fifteen predictors that impacted model accuracy most, by calculating the mean decrease in accuracy that would result from excluding each predictor from the model. The goal of this exercise is to gain insight into what patient factors are the most promising targets of prevention strategies.

## Results

### Descriptive statistics

The sample used for predicting AMI (model 1) included 13,079 patients of whom 59.3% had the male sex (Table 1). The mean age of the cohort was 70.6 (SD 11.5) years. In total, 16.8% (n = 2189) of patients in this sample suffered an AMI. The sample for model 2 (predicting IHD) included 13,218 patients of which 59.2% had the male sex. The mean age was 70.7 (SD 11.1) years and 16.2% (n = 2139) suffered from IHD.

### Model performance

#### Prediction of acute myocardial infarction

Table 2 shows the models’ skill based on the six cross-validated performance metrics. For the ML model predicting AMI, the accuracy was 0.96, sensitivity was 0.67, and specificity and precision were both 1.00. The AUC was 0.96 and the Brier score 0.04.

**Fig 2:**
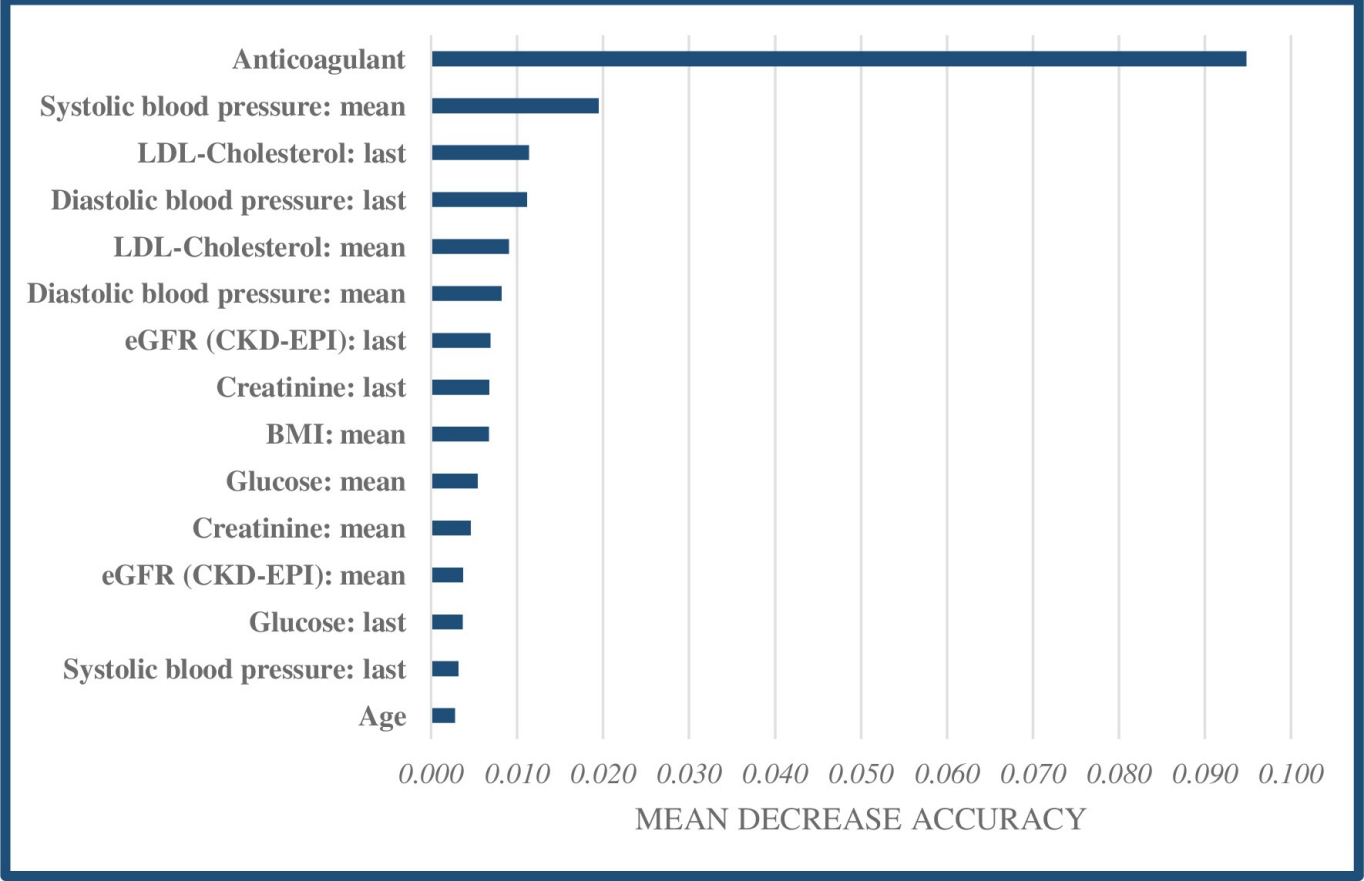
Top 15 predictors in the ML model for AMI based on mean decrease accuracy.

Fig 2 presents an overview of the fifteen predictors that impacted model accuracy most. These predictors are ranked based on the mean decrease in accuracy that would occur if each predictor would be excluded from the model (note that the figure does not indicate whether each variable had a positive or negative association with the outcome). The use of anticoagulants/antiplatelets stands out, which contributed 0.095 to the accuracy of the model. Several biomarkers are also important for model accuracy, especially mean systolic blood pressure, last measurement of LDL-cholesterol, mean LDL-cholesterol, and last measurement of diastolic blood pressure.

#### Prediction of ischemic heart disease

As shown in Table 3, the ML-model for IHD had an accuracy of 0.96, sensitivity of 0.68, specificity of 1.00, and precision of 1.00. The AUC was 0.96 and the Brier score 0.03.

**Table 3:** Cross-validated performance metrics for models predicting IHD (mean of folds).

Performance metric	Machine learning model 2	SMART 2
Accuracy	0.96	0.71
Sensitivity	0.68	0.02
Specificity	1.00	0.84
Precision	1.00	0.03
AUC/c-statistic	0.96	0.43
Brier-score	0.03	0.29

**Fig 3:**
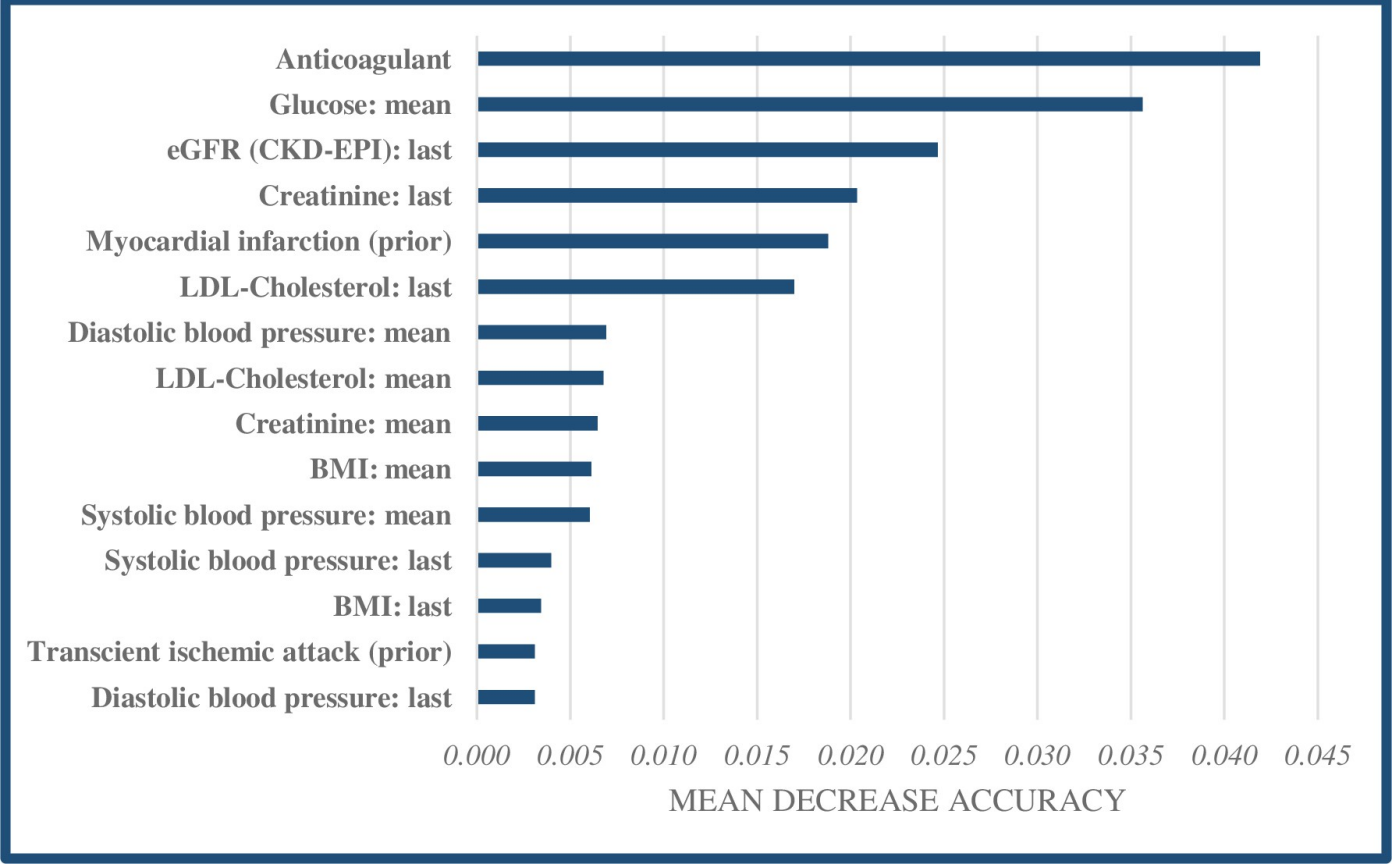
Top 15 predictors in the ML model for IHD based on mean decrease accuracy.

Like the AMI prediction model, the variable anticoagulants/antiplatelets had the most significant impact on the accuracy of the IHD model, although the contribution of this predictor was smaller (0.04) compared to the AMI model (Fig 3). Aside from anticoagulants/antiplatelets, the variables that had the greatest influence on model accuracy were blood glucose level, the most recent eGFR, the most recent creatinine measurement, and prior myocardial infarction. For IHD, performance was slightly better but similar overall.

#### Predictions based on the SMART algorithm

The same datasets used for the ML prediction models for AMI and IHD were also used to evaluate the performance of the SMART algorithm. In both cases, the SMART algorithm showed poorer performance than the ML models (Tables 2, 3). For the AMI prediction, for example, the accuracy of the SMART algorithm was 0.67, sensitivity was 0.03, specificity was 0.80, precision was 0.03, AUC was 0.42, and the Brier score was 0.33.

## Discussion

### Summary and discussion of main findings

In this study, we aimed to predict AMI and IHD in primary care cardiovascular patients using machine learning (ML). We evaluated the predictive performance of two random forest models and made a head-to-head comparison with the commonly used SMART algorithm. The results indicate that given the data, ML can accurately predict whether patients will or will not develop AMI or IHD. The model for AMI had a sensitivity of 68% (i.e., this model correctly predicts around seven out of ten AMIs) and a specificity of 100% (i.e., nearly all patients without AMI were identified correctly as such), with excellent discrimination (AUC), calibration (Brier score) and accuracy (i.e., nearly all patients who are predicted to suffer an AMI indeed suffered from one). Performance metrics for the IHD prediction model were slightly lower, but overall similar. By contrast, performance of the SMART algorithm on the same populations was substantially lower, for both AMI and IHD. This suggest that ML may be more appropriate for predicting CVD than the existing SMART algorithm, although the question remains whether the superior performance of our ML models would also be achieved when the available set of predictors is less extensive. Regardless of this comparison, the good performance of the ML models underscores the potential of using ML for CVD prediction purposes in primary care settings.

Several reasons may underly the underperformance of the SMART algorithm relative to our ML models. First, the SMART algorithm was originally developed to estimate long-term risk of vascular events [17]. To enable direct comparison of performance with our ML models, we applied the SMART algorithm to a binary dependent variable (i.e., yes/no AMI or IHD). This might have caused some underperformance. Second, The SMART algorithm was initially tested and validated in a hospital setting instead of in a primary care setting [48]. Given the lack of available prediction models validated specifically for primary care cardiovascular patients in the context of secondary prevention, the SMART algorithm was the closest benchmark for making comparisons in the context of this study.

Ideally, the performance of our ML models is best compared to prediction models that were developed under similar conditions, that is, using data from a primary care setting including data on similar patients and focusing on predicting the same events (i.e., AMI or IHD). Although many ML prediction models have been developed for CVD, very few meet each of these conditions, especially due to a lack of models that were developed in a primary care setting. An exception are the models (i.e., Neural Network, Random Forest, Logistic Regression, Gradient Boosting) developed by Weng et al. (2017) who aimed to predict ‘fatal or non-fatal’ CVD [37]. Interestingly, both of our ML models had substantially better performance in terms of AUC, sensitivity, and specificity (other measures were not reported). Possible explanations for this difference in performance are differences in the included population (i.e., all primary care patients in Weng et al. versus cardiovascular patients only in our study), lack of imputation in Weng et al., and the fact that we were able to include more predictors (e.g., biometrics, existing comorbidity).

Our ML model for IHD generally also showed better performance compared to the ML models (Back-propagation neural network (BPNN) and Bayesian neural network (BNN)) developed by Kangwanariyakul and colleagues, who also aimed to predict IHD [49]. Although that study did focus on CVD patients (albeit in a hospital setting, which likely means a higher a-priori probability of cardiovascular events than in a primary care setting), the authors used magneto-cardiogram data for their predictions which will typically be unavailable in a primary care setting.

Furthermore, our models generally showed better performance than previously developed models that tried to predict CVD in general [14, 15, 17, 36, 37, 48, 50, 51]. Having specific predictors that are particularly pertinent to a particular event can result in superior model performance in predicting that event compared to applying the model to a broader population, and vice versa. By tailoring the model to a specific group of patients, we can potentially enhance its performance and increase the relevance for clinical practice [17, 36, 48].

### Implications

Our findings have several implications and could aid clinical decision-making in multiple ways. First, a recently published validation study on predicting event rates established that the SMART algorithm shows similar retrospective and prospective performance [36]. As such, the SMART algorithm has the potential to support personalized, well-informed, and collaborative decision-making on treatment strategies, particularly in cases where costly interventions may only benefit specific patients in secondary prevention. Given that our ML models outperform the SMART algorithm in predicting CVD events and shows substantially better performance than the metrics reported in the validation study (although the patient cohort in that study was larger and somewhat younger on average than in our study), this underlines the potential of using ML in supporting cardiovascular risk management [52]. Therefore, healthcare professionals, patients, and researchers might benefit from adopting ML models for CVD risk prediction [52]. One major advantage of our models is that they do not strictly rely on separate independent variables. In traditional modelling approaches, individual risk factors like cholesterol levels or BMI are considered independently. In contrast, ML can capture interactions between these factors. For instance, patients may experience significant benefits from risk reduction strategies only when both cholesterol and BMI are simultaneously lowered. Thus, ML models offer the potential for improved CVD risk prediction by considering the complex interactions among multiple factors [53, 54], which in turn could provide more accurate risk assessments and result in better-informed decision-making (it is important to acknowledge, however, that this does not necessarily always hold since there are ML models specifically designed to excel when individual variables are treated independently in different contexts). Moreover, given their high sensitivity and specificity, our ML models may help in reducing unnecessary hospital referrals and thereby the burden on patients and the healthcare system. The high specificity suggests that interventions for healthy individuals can be avoided while the high sensitivity indicates that at the same time the risk that individuals who truly have the condition are not referred is minimized.

Third, although our ML models do not provide insight into the sign of the relationships between the predictor variables and outcomes (i.e., positive, or negative), it was possible to identify the most influential predictors. Anticoagulants/antiplatelets and several biomarkers, including blood pressure measurements and LDL-cholesterol levels, were found to be important in accurately predicting cardiovascular risk. Therefore, primary care providers should pay extra attention to these specific predictors in cardiovascular patients, although it is important to emphasize that these predictors may not only impact the outcomes independently but are also like to interact, influencing overall risk. Additional research is necessary on how predictions of CVD events can contribute to improving guidelines that aim for secondary prevention of CVD through (timely) risk identification. Ideally, this would occur through a randomized controlled trial in which patient outcomes are compared between GPs that are providing care based on the results of prediction models and GPs providing usual care [53, 54].

Finally, although the accessibility of prediction models in healthcare is improving, they require specific expertise to develop and use [55]. Yet primary care practices typically lack the necessary expertise and resources for that. This holds especially for AI-based prediction models [52]. More centralized development of prediction models should be considered as this could result in better accessibility, more efficiency, higher patient volumes, and lower administrative burden. Such centralisation would however require substantial investment in the primary care data infrastructure in many countries (see also below).

### Strengths and limitations

A major strength of this study is the head-to-head comparison with an existing risk prediction model for CVD. Another strength is that our ML models were developed using data from a primary care setting, where relative to secondary care much effort is being put in (secondary) prevention. This contrasts with previously developed ML models, which are often based on data from hospital settings. Other strengths include the large sample size and the use of recent data over a 10-year period. Results are therefore likely to be representative for a sample of primary care cardiovascular patients we have today. Finally, the use of cross-validation and imputation helped us to address overfitting and missing values.

However, several limitations should also be mentioned. First and foremost, although our models show high predictive skill and ML in general is capable of handling complex data, ML comes with difficulty in terms of the interpretability of the effects of individual predictors and the interaction between them. Although we have provided some insight into this ‘black box’ by identifying the most influential patient factors based on the mean decrease in accuracy (providing some insight into what patient factors could be targeted to mitigate cardiovascular risk), individual effects of predictors remain difficult to interpret. However, the patient factors and subsequent interventions identified as most important for the accuracy of the models (e.g., use of anticoagulant/antiplatelet drugs, managing blood pressure, managing LDL cholesterol, and eGFR) do offer GPs and patients actionable tools to mitigate cardiovascular risk. While it is complex to predict the exact degree to which this risk can be mitigated, we believe it is worthwhile to pursue these interventions when the model classifies a patient as ‘high-risk’.

Relatedly, we acknowledge that factors unavailable in our data, such as genetics, could strongly influence outcomes. Nevertheless, this does not take away the fact that available risk factors can also have strong predictive capabilities. ML is likely to perform well when prediction is the primary goal, but effective cardiovascular risk management also requires insight into what specific patient factors to focus on and why. Relatedly, despite using a large dataset and temporal cross-validation, we acknowledge as a limitation the potentially limited generalizability of our results to other settings, populations and timeframes. A second limitation is that although we internally validated our models, external validation is needed to assess how the models would perform in different populations. Given the relatively large number of GPs in the dataset, we believe the risk of bias arising from this factor to be low. Regarding the timeframe related to the data, to prevent overfitting and mitigate potential time-dependent bias, we employed temporal cross-validation. This method is particularly useful when dealing with time-dependent health data, as is the case with our data. However, we acknowledge that we cannot formally exclude the possibility that this may have influenced our results.

Third, we acknowledge the potential differences in the risk profiles of patients with only risk factors compared to those with existing (atherosclerotic) CVD. However, in practice, these patients are often grouped together in cardiovascular risk management programs based on having risk factor(s) that are commonly associated with CVD. Our dataset comprises patients enrolled in such programs, emphasizing a practical perspective that places our study within the context of general prevention in primary care for populations at risk of severe CVD. Although the focus of our study was therefore not solely on primary or secondary prevention, we believe that understanding CVD event rates through prediction modelling is valuable for informing clinical decisions, particularly for patients in primary care cardiovascular risk management programs [56, 57]. Personalized risk predictions can empower patients to adopt healthier lifestyles, adhere to medications, and actively participate in preventive measures [58, 59]. Nevertheless, we recognize that focussing on risk prediction in the context of primary prevention would also be an interesting avenue for future research. A final and related limitation is that our results are conditional on patients enrolled in a CVRM program. This means that these individuals are already under monitoring and participating in a preventive program aimed at reducing the risk of cardiovascular disease, whereas the greatest potential for improving public health lies in reaching out to those patients for primary prevention. Finally, further studies on predicting CVD with ML in primary care in other settings, populations and timeframes are needed to validate our results in order to provide better and more generalized tools for GPs and patients to manage CVD risk. Moreover future studies should focus on developing more explainable algorithms to further enhance the interpretability of machine learning models, particularly in clinical contexts where transparency in decision-making is paramount.

## Conclusions

Our ML models showed high predictive performance and outperformed the existing SMART algorithm in predicting AMI and IHD in primary care cardiovascular patients. This underlines the potential of using ML for CVD prediction purposes in primary care settings. Although in this respect ML models seems promising for cardiovascular risk prediction, interpretability of the (interacting) effects of predictor variables remain an issue. Nonetheless, primary care providers, patients, and researchers may benefit from transitioning towards using ML models for support of individualized predictions and subsequent (secondary) prevention in primary care cardiovascular patients.

## Supporting information

S1 Appendix International classification of primary care (ICPC) codes. (DOCX)

S2 Appendix Included pulmonary asthma medications. (DOCX)
