# Supplementary material for: Using machine learning to predict acute myocardial infarction and ischemic heart disease in primary care cardiovascular patients
Source: PLoS One. 2024 Jul 18;19(7):e0307099. doi: 10.1371/journal.pone.0307099 (PMC11257251; doi:10.1371/journal.pone.0307099)
Supplement: S1 Appendix — (DOCX) [file pone.0307099.s001.docx]

**Appendix 1 – International Classification of Primary Care (ICPC) codes**

**ICPC codes that are commonly associated with cardiovascular disease**

**(inclusion criteria for existing cardiovascular disease, included patients should have at least one):**

K74: Ischemic heart disease with angina

K75: Angina pectoris

K76: Acute myocardial infarction

K77: Chronic ischemic heart disease

K78: Heart failure

K79: Rheumatic heart disease

K80: Cardiac arrhythmias

K81: Hypertensive heart disease

K82: Other heart disease

K83: Acute cerebrovascular disease

K84: Chronic cerebrovascular disease

K85: Peripheral arterial disease

K86: Aortic aneurysm/dissection

K87: Venous thrombosis/embolism

K88: Other vascular diseases

K89: Hypertension

K90: Other circulatory system disorders

K91: Stroke

K92: Transient ischemic attack (TIA)

K93: Haemorrhagic cerebrovascular disease

K94: Pulmonary embolism

K95: Pulmonary hypertension

K96: Deep vein thrombosis

K99: Other cardiovascular diseases

**ICPC code inclusion acute myocardial infarction:** K76: Acute myocardial infarction

**ICPC codes inclusion symptomatic ischemic heart disease:** K74: Ischemic heart disease with angina
