# Supplementary material for: Using machine learning to predict acute myocardial infarction and ischemic heart disease in primary care cardiovascular patients
Source: PLoS One. 2024 Jul 18;19(7):e0307099. doi: 10.1371/journal.pone.0307099 (PMC11257251; doi:10.1371/journal.pone.0307099)
Supplement: S2 Appendix — (DOCX) [file pone.0307099.s002.docx]

**Appendix 2- Included pulmonary asthma medications**

| Fluticasone |
| --- |
| Salbutamol Inhalation |
| Tiotropium |
| Salmeterol/Fluticasone |
| Tiotropium/Olodaterol |
| Ipratropium |
| Beclomethasone |
| Formoterol/budesonide |
| Aclidinium/Formoterol |
| Formoterol/Beclomethasone |
| Glycopyrronium Inhalation |
| Salmeterol |
| Budesonide |
| Terbutaline |
| Ciclesonide |
| Fenoterol/Ipratropium |
| Formoterol |
| Formoterol/budesonid |
| Salbutamol/ipratropium |
| Aclidiniumbromid |
| Indacaterol |
| Olodaterol |
| Vilanterol/fluticasonfuroate |
| Beclomethasone/formoterol/glycopyrronium |
| Fluticasone/umeclidinium/vilanterol |
| Umeclidinium/vilanterol |
| Umeclidinium |
| Formoterol/fluticasone |
| Glycopyrronium/formoterol |
| Cromoglicine acid |
| Formoterol/glycopyrroniumbromide |
| Budesonide/salmeterol |
| Nedocromil |
| Flunisolide |

Table 1. Included pulmonary asthma medications.

**Included anticoagulants and antiplatelet medications.**

| fenprocoumon |
| --- |
| acenocoumarol |
| heparin |
| dalteparin |
| enoxaparin |
| nadroparin |
| clopidogrel |
| Aspirin |
| dipyridamol |
| Carbasalate calcium |
| prasugrel |
| ticagrelor |
| selexipag |
| dabigatranetexilaat |
| Direct factor Xa inhibitors |
| rivaroxaban |
| apixaban |
| edoxaban |
| fondaparinux |

Table A2. Included anticoagulants and antiplatelets.
